# N^6^-Methyladenosine Associated Silencing of miR-193b Promotes Cervical Cancer Aggressiveness by Targeting CCND1

**DOI:** 10.3389/fonc.2021.666597

**Published:** 2021-06-10

**Authors:** Chunxian Huang, Jinxiao Liang, Shaodan Lin, Dongyan Wang, Qingsheng Xie, Zhongqiu Lin, Tingting Yao

**Affiliations:** ^1^ Department of Gynecological Oncology, Sun Yat-Sen Memorial Hospital, Sun Yat-Sen University, Guangzhou, China; ^2^ Guangdong Provincial Key Laboratory of Malignant Tumor Epigenetics and Gene Regulation, Sun Yat-Sen Memorial Hospital, Sun Yat-Sen University, Guangzhou, China

**Keywords:** m^6^A, METTL3, miR-193b, CCND1, cervical cancer

## Abstract

**Objective:**

Cervical cancer is a frequently encountered gynecological malignancy as a major contributor to cancer-related deaths in women. This study focuses on how miR-193b promotes cervical cancer aggressiveness as well as the role of m^6^A in miR-193b silencing.

**Methods:**

Cervical cancer samples and the matching adjacent normal cervical tissues were used to determine the significance of miR-193b in cervical cancer. The CCK-8 assay, cell cycle analysis, qRT-PCR, Western blot assay, IHC, RIP, and xenograft models were utilized to explore the impact of miR-193b in cervical cancer and how m^6^A regulates miR-193b expression. Luciferase reporter assays, qRT-PCR, and Western blotting were enlisted to study the interaction between miR-193b and CCND1.

**Results:**

Our study suggested that lower miR-193b expressions were strongly linked to more advanced cervical cancer stages and the presence of deeper stromal invasion. miR-193b functions as a tumor suppressor that is regulated by m^6^A methylation in cervical tumors. METTL3 modulates miR-193b mature process in an m^6^A-dependent manner. Reintroduction of miR-193b profoundly inhibits tumorigenesis of cervical cancer cells both *in vivo* and *in vitro* through CCND1 targeting.

**Conclusions:**

m^6^A associated downregulation of miR-193b promotes cervical cancer aggressiveness by targeting CCND1.

## Introduction

Cervical cancer is a frequently encountered gynecological malignancy as a primary cause of cancer-associated mortality in women (1). Nearly 90% of deaths occurred in developing countries, and more than half of these were recorded in Asia (2). Most patients respond to standard treatments which include surgery and radiotherapy. However, there remains a substantial risk of tumor relapse in patients who develop locally advanced cervical cancer with high risks such as positive lymph nodes or positive surgical margins. Among these groups of patients, relapse rates exceeding 50% after standard treatments have been documented. 5-year survival rates upon relapse remain dismal at only 30–60% (3, 4). Therefore, the identification of novel therapeutic methods is critical, and inevitably requires a deeper grasp of cervical cancer biology.

Gene expression has been noted to be strongly influenced by RNA modification, thereby leading to the development of the new field of RNA epigenetics (5). One ubiquitously occurring epigenetic modification is mediated by N^6^-methyladenosine (m^6^A), which methylates the adenosine N^6^-position in mammalian mRNA (6). m^6^A is necessary for a myriad of physiological processes, such as cell status regulation, sex determination, and development (7–9). Accumulating evidence indicates that m^6^A regulates RNA metabolism, including mRNA translation, degradation, splicing, export, and folding. Recent studies have uncovered an important post-transcriptional effect of m^6^A, which is the triggering of microRNA (miRNA) biogenesis (10). Alteration of m^6^A level was found across several cancers such as glioblastoma (9), lung cancer (11), gastric cancer (12), and hepatocellular carcinoma (13). Dysregulated m^6^A modification affects cancer pathogenesis and development by modulating the expression of tumor-related genes such as EGFR, BRD4, SOCS2, and MYC (14).

miRNAs are small non-coding RNAs (18-24 nucleotides) that usually negatively modulate genetic expression at the post-transcriptional stage by forming bonds to their target mRNA at the 3’-untranslated region (UTR) (15). Numerous physiological as well as pathophysiological processes, including carcinogenesis, are influenced by miRNA activity (16, 17). In this study, we found that miR-193b acts as a tumor suppressor in cervical cancer. A combination of bioinformatics algorithms, functional analyses, and xenograft models revealed the pivotal roles of METTL3/miR-193b/CCND1 signaling in cervical cancer aggressiveness.

## Materials and Methods

### Patients and Tissue Samples

41 pairs of cervical cancer tissues and their surrounding control tissue specimens were obtained from Sun Yat-Sen Memorial Hospital (Guangzhou, China) after surgical resection. Clinicopathological characteristics included FIGO stage, tumor grade, histologic subtype, invasion depth, lymph node metastasis, lymphatic vascular space invasion, and age were collected. Approval from the Research Ethics Committee of Sun Yat-Sen Memorial Hospital was obtained before the study. The research was performed following concepts established by the Declaration of Helsinki.

### Cell Culture

Siha and Hela cell lines (human cervical cancer cell lines) were procured from the Cell Bank of the Chinese Academy of Sciences (Shanghai, China) and tested by STR Authentication. Cells were cultured in DMEM (Gibco, USA) supplemented with 10% fetal bovine serum (Biological Industries, Israel). Cultures were maintained in an incubator at 37°C with a humidified 5% CO_2_ environment.

### RNA Extraction and qRT-PCR

The Trizol reagent (Takara, Japan) was utilized for RNA extraction followed by the use of the PrimeScript RT reagent kit (Takara, Japan) for synthesizing cDNA. Experiments were in strict compliance with protocols published by the manufacturer. miRNA and mRNA relative expression levels were confirmed using qRT-PCR as described before (18). U6 and GAPDH were used for normalization.

### Cell Transfection

The sequences of miR-193b mimic (miR-193b), miRNA negative control, inhibitor targeting miR-193b (anti-miR-193b), corresponding negative control, siRNA METTL3, and siRNA negative control were listed in **Supplementary Material**. LipofectamineTM2000 (Invitrogen, USA) was used in cell transfection following instructions stipulated by the manufacturer.

### Cell Proliferation Assay

The Cell Counting Kit 8 (CCK8) (Dojindo, Japan) was used to assess cell proliferation. Cells were incubated with CCK8 at a final concentration of 100 μl/ml for 1.5 hours at 37°C. Absorbance was recorded at 450 nm, with the absorbance at each time point used to construct cell proliferation curves.

### Cell Cycle Analysis

Cells were fixed in 70% cold ethyl alcohol for 30 min at 4°C. 100 μg/ml RNase and 0.1 mg/ml propidium iodide were subsequently added. The distribution of cell cycle phases was determined by the Cell Quest software (BD Biosciences, USA).

### Xenograft Models and Treatment

Female nude mice of ages 4 weeks were purchased from the Sun Yat-Sen University Laboratory Animal Center (Guangzhou, China) and treated according to the National Institutes of Health guide for the care and use of laboratory animals. All animal experimental protocols were approved by the Animal Research Committee of Sun Yat-Sen University. Mice were divided into two groups (n = 4/group) randomly. 3×10^6^ cells suspended in 200 μl PBS were administered *via* subcutaneous injection over the right flank region of nude mice. After the development of palpable tumors (average volume, 50 mm^3^), intratumoral injection of synthetic miR-193b, or negative control complexed with siPORT Amine transfection reagent (Ambion, USA) was given 6 times at a 4-day interval. Animals were sacrificed and tumors were harvested 24 days after the first injection. Tumor volumes derived as follows: V = (L × W2)/2 (L: length, W: width).

### Immunohistochemistry (IHC)

5 μm cervical cancer paraffin-embedded tissue sections were subjected to xylene paraffin removal followed by ethanol rehydration. Freshly prepared 3% hydrogen peroxide was used to inhibit the activity of endogenous peroxidases. Sections were then incubated overnight with anti-ki-67 (Cell Signaling Technology, USA) at 4°C, rinsed thrice with PBS, and incubated with anti-rabbit & mouse IgG (DAKO, Denmark) at room temperature for 1 hour. Diaminobenzidine (DAKO, Denmark) was used to visualize the bound antibody.

### Western Blot Analysis

Protein was harvested using lysis buffer (CWBIO, China) containing a protease inhibitor cocktail (Roche, Switzerland). 25 ug protein were separated by 10% SDS-PAGE electrophoresis and immunoblotted onto a PVDF membrane (Bio-Rad, USA). Specific primary antibodies were then incubated with the samples at 4°C for 12-16 hours after blocking endogenous reactions. Following a one-hour incubation with secondary antibodies at room temperature the next day, bound proteins were imaged with an enhanced chemiluminescence detection kit (Millipore, USA). Primary antibody for METTL3, CyclinD1, internal reference antibody for β-actin, HRP Goat-anti-Rabbit, and HRP Goat-anti-Mouse were obtained from Cell Signaling Technology (USA).

### Luciferase Reporter Assay

Mutant (MUT) and wild-type (WT) CCND1 3’-UTR which contained predicted miR-193b target sites were amplified by the PCR method. Primers for reporter vector construction were given in **Supplementary Material**. WT or MUT reporter plasmid and miR-193b or miRNA negative control were transfected into HEK-293T cells. Firefly luciferase activities were assessed 24 hours post-transfection with the Dual Luciferase Assay (Promega, USA). Renilla luciferase was used to normalize results.

### Measurement of m^6^A Modification

After extracting total RNA from each group using the Trizol reagent (Takara, Japan), the m^6^A content was measured by using a commercial kit (Abcam, USA). Briefly, capture antibody solution and detection antibody solution were then added in assay dishes, where 200 ng RNAs were plated on. Absorbance was recorded at 450 nm. The m^6^A levels were quantified by the absorbance of each sample.

### RNA Immunoprecipitation (RIP)

RIP experiments were carried out using the Magna RIP Kit (Millipore, USA) according to the instructions stipulated by the manufacturer. Briefly, cells were isolated and treated with deoxyribonuclease I, and then fragmented by sonication. IPs were performed with an anti-m6A antibody. RNAs were extracted and subjected to qRT-PCR using primer of pri-miR-193b and finally normalized to input.

### Statistical Analysis

The SPSS 20.0 software was enlisted to carry out all statistical analyses. Both the two-tailed Chi-square test and Student’s t-test were used. Statistical significance was determined by the finding of *p* < 0.05. All experiments were repeated independently in triplicate.

## Results

### Determination of the Clinical Significance of miR-193b in Cervical Cancer

qRT-PCR was used to determine miR-193b levels in clinical cervical cancer specimens and the matching surrounding normal cervical tissues (N = 41). 26 of these tumor samples (63%) contained low miR-193b levels. These findings indicate that miR-193b suppression may be a key feature leading to the progression of human cervical cancer. Notably, lower miR-193b levels were linked to a more advanced FIGO stage (*p* = 0.031) and deeper stromal invasion (*p* = 0.006) (**Table 1**). However, there was no significant correlation between the low level of miR-193b and tumor grade, histologic subtype, lymphatic vascular space invasion, or lymph node metastasis (**Table 1**). These results show that miR-193b silencing played a crucial part during cervical cancer development.

**Table 1:** Clinical Significance of miR-193b in cervical cancer.

Variable	Cases		High miR-193b expression		Low miR-193b expression		*p* value^a^
	N	(%)	N	(%)	N	(%)	
Age (years)^b^							
<50	23	56.10	11	47.83	12	52.17	0.091
≥50	18	43.90	4	22.22	14	77.78	
FIGO stage							
<Ib3	21	51.22	11	52.38	10	47.62	0.031
≥Ib3	20	48.78	4	20.00	16	80.00	
Histologic subtype							
Squamous	27	65.85	11	40.74	16	59.26	0.132
Non-squamous	14	34.15	4	28.57	10	71.43	
Tumor grade							
G1/G2	23	56.10	7	30.43	16	69.57	0.355
G3	18	43.90	8	44.44	10	55.56	
Depth of invasion							
>1/3	25	60.98	5	20.00	20	80.00	0.006
≤1/3	16	39.02	10	62.50	6	37.50	
Lymphatic vascular space invasion							
Positive	19	46.34	5	26.32	14	73.68	0.205
Negative	22	53.66	10	45.45	12	54.55	
Lymph node metastasis							
Positive	10	24.39	2	20.00	8	80.00	0.147
Negative	31	75.61	13	41.34	18	58.06	

a χ^2^ test.

b Range 19-79 years, median 50 years.

### miR-193b Is a Tumor Suppressor in Cervical Cancer

**Figure 1:**
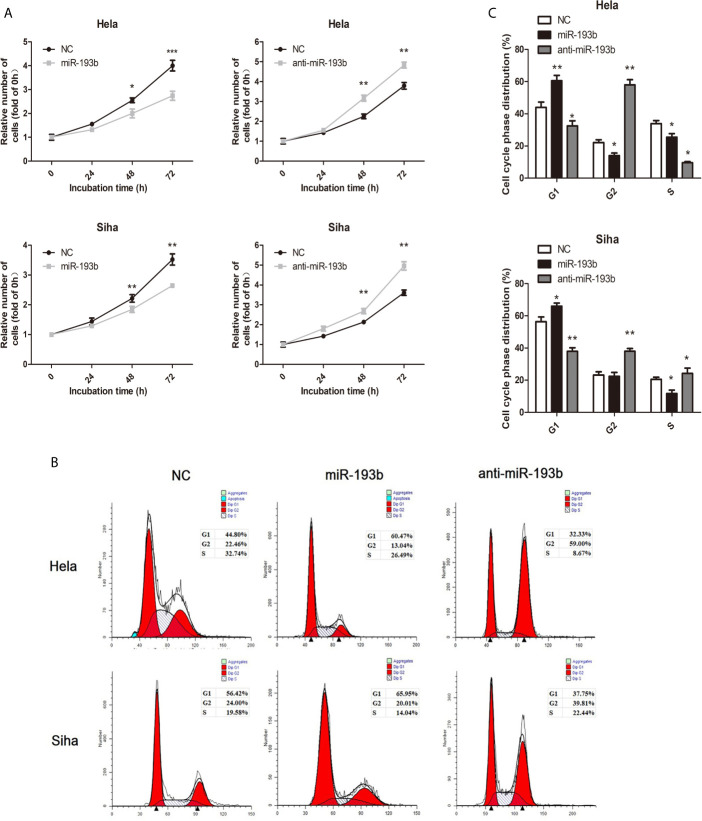
miR-193b is a tumor suppressor in cervical cancer. Either miR-193b or anti-miR-193b was used to transfect Hela and Siha cells as described. **(A)** CCK-8 assays were carried out to determine cellular proliferation. **(B)** Flow cytometry was used to assess distributions of cells across the cell cycle. **(C)** The qualifications of the cell cycle were presented. *p < 0.05, **p < 0.01, ***p < 0.001.

We investigated miR-193b function by introducing miRNA or anti-miRNA into Hela and Siha cells. The efficiency of transfection was verified by qRT-PCR (**Supplementary Figure 1**). Reintroduction of miR-193b could markedly inhibit cell proliferation in cervical cancer (**Figure 1A**), while the addition of a miR-193b inhibitor (anti-miRNA) nullified this phenomenon (**Figure 1A**). Cell cycle assays showed that miR-193b mimics induced increases in the G2 population (**Figures 1B, C**), implying G2 arrest. As expected, miR-193b inhibitor significantly promoted cell cycle progression from G0–G1 into S and G2-M phase (**Figures 1B, C**). These results further strengthen our hypothesis of miR-193b as a tumor-suppressing molecule in cervical cancer.

### miR-193b Suppresses *In Vivo* Cervical Cancer Growth

**Figure 2:**
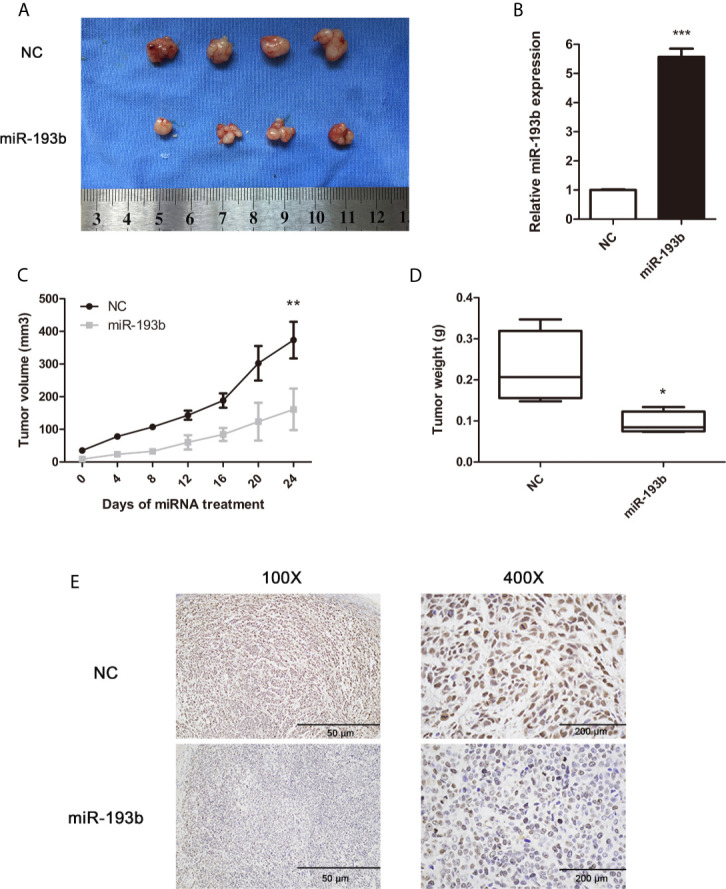
miR-193b suppresses cervical cancer growth *in vivo*. Subcutaneous Siha tumors located on mice xenograft models were directly injected with miR-193b for a total of 6 times at 4-day intervals. Tumors were harvested on the 24th day from the injection. **(A)** miR-193b inhibited cervical cancer growth *in vivo*. **(B)** miR-193b expression levels of each group after intratumoral miR-193b injections were measured by qRT-PCR. **(C)** Effect of miR-193b on tumor growth and tumor volumes. **(D)** The tumors were weighed after harvested. **(E)** The protein levels of Ki-67 were detected using IHC. *p < 0.05, **p < 0.01, ***p< 0.001.

To determine the *in vivo* antitumor miR-193b impact, we first induced the formation of Siha-bearing tumors in mice. These xenograft tumors were then injected directly with the relevant miRNAs. miR-193b levels increased by 5.7 fold after this treatment (**Figure 2B**). After 3 weeks, miR-193b treated tumors grew more slowly than those receiving negative control miRNA injections (**Figures 2A, C**). We also noted a significant difference in tumor weights at the end of the treatment protocol (**Figure 2D**). Ki-67 staining was significantly reduced after miR-193b treatment (**Figure 2E**). These results suggest that miR-193b suppresses cervical cancer growth in the xenograft models.

### miR-193b Regulates the Expression of CCND1

**Figure 3:**
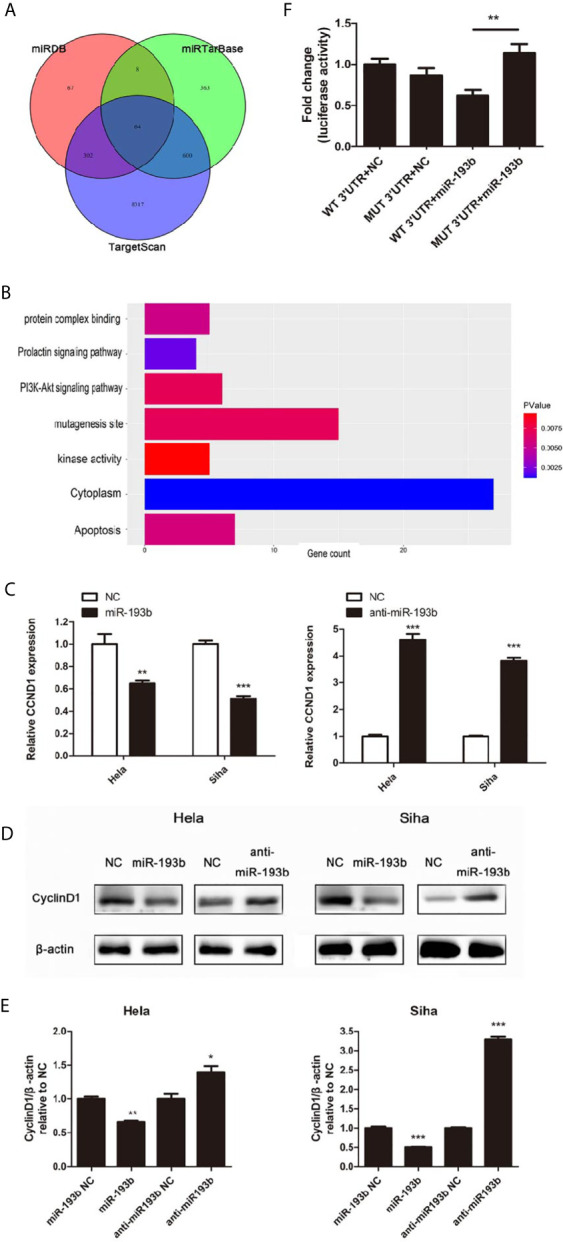
miR-193b regulates the expression of CCND1. Either miR-193b or anti-miR-193b was used to transfect Hela and Siha cells as described. **(A)** Venn diagrams showed 64 significantly target genes that appeared simultaneously in miRNA target prediction algorithms TargetScan, miRDB, and miRTarBase. **(B)** GO analysis was performed on 64 targeted genes. **(C)** CCND1 expressions were measured by qRT-PCR. **(D)** CyclinD1 protein expression was detected using Western blot. **(E)** Band density of Western blot was examined using ImageJ, and protein levels were normalized to β-actin. **(F)** 3′-UTR luciferase reporter assay was utilized to determine the binding of miR-193b to the 3′-UTR of CCND1. Luciferase activities of WT or MUT CCND1 3′-UTR luciferase construct co-transfected with miR-193b or NC in HEK-293T cells were measured. *p < 0.05, **p < 0.01, ***p < 0.001.

Further experiments were carried out to discern the effect of miR-193b silencing on its initially unknown target gene. miRNA target prediction algorithms TargetScan, miRDB, and miRTarBase were used to identify the target genes of miR-193b. Venn diagrams showed 64 significantly target genes that appeared simultaneously in three prediction algorithms (**Figure 3A**). Gene ontology (GO) analysis was performed on 64 targeted genes and the enriched pathways were mainly associated with responses to apoptosis, protein complex binding, prolactin signaling pathway, PI3K-Akt signaling pathway, mutagenesis site, kinase activity, and cytoplasm (**Figure 3B**). CyclinD1 (CCND1) was a vital gene among the 64 predicted target genes given previous studies alluding to its importance in cervical cancer development (19, 20). As one of the cell cycle regulators, CCND1 is upregulated in cervical cancer and has a crucial role in enhancing tumorigenesis (21–24). CCND1 was highly expressed in low miR-193b level patients (**Table 2**). CCND1 expression was inhibited in Hela and Siha cells which overexpressed miR-193b, however, the converse was seen in cells with suppressed miR-193b levels (**Figure 3C**). A similar result was noted in CyclinD1 protein expression (**Figures 3D, E**), confirming that miR-193b regulated CCND1 mRNA expression in cervical cancer cells. We further sought to determine the direct miRNA binding site on CCND1 3′-UTR through luciferase reporter assays using WT and MUT miR-193b seed matched human CCND1 3′-UTR constructs. Co-transfection of miR-193b and the WT construct diminished luciferase activity. This effect was not seen with the use of the MUT construct, demonstrating that CCND1 was a direct target of miR-193b (**Figure 3F**). All the findings above suggest that downregulating miR-193b resulted in CCND1 modulation.

**Table 2:** Relation between miR-193b, and CCND1 in cervical cancer.

Variable	Cases		High miR-193b expression		Low miR-193b expression		*p* value^a^
	N	(%)	N	(%)	N	(%)	
CCND1							
High expression	21	56.1	4	9.76	17	41.46	0.017
Low expression	20	75.61	11	26.83	9	21.95	

a χ^2^ test.

### miR-193b Is Regulated by m^6^A Methylation

**Figure 4:**
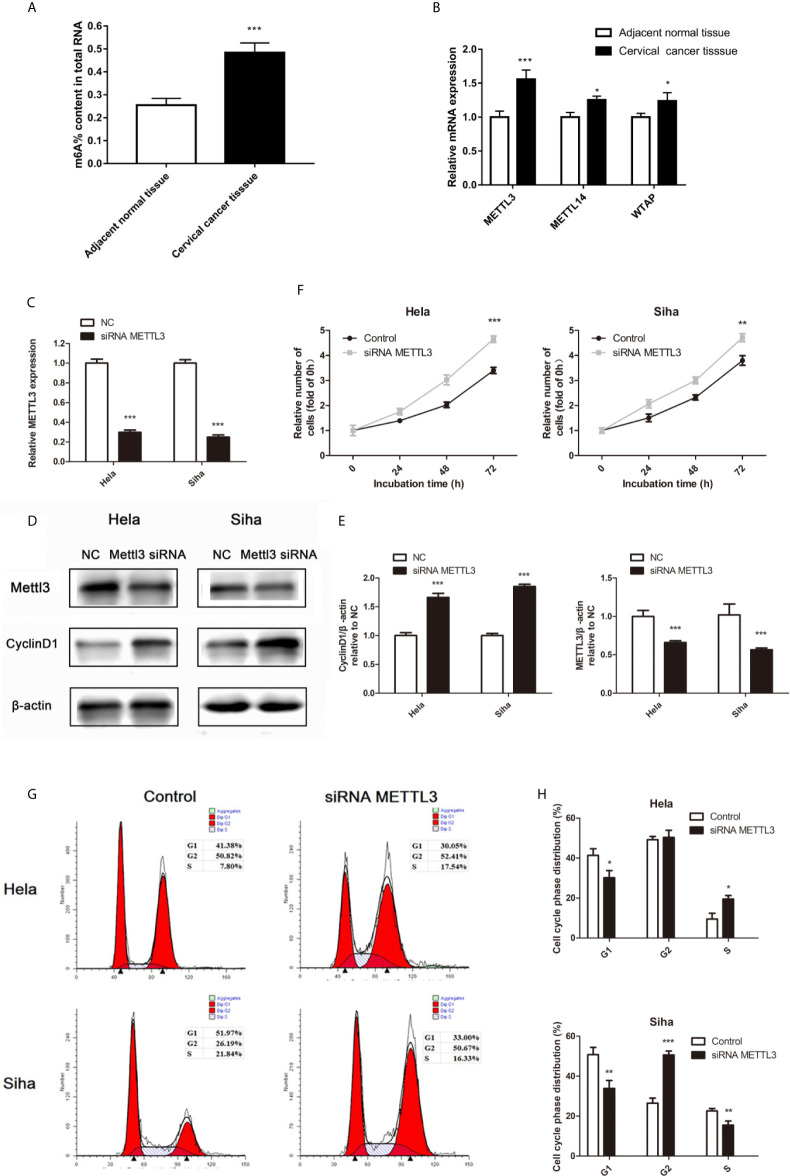
m^6^A methylation in cervical cancer. **(A)** The total m^6^A methylation level in cervical cancer tissues and adjacent normal tissues. **(B)** mRNA expression of METTL3, METTL14, and WTAP were measured by qRT-PCR. **(C–E)** Hela and Siha cells were transfected with siRNA METTL3 or negative control. METTL3 mRNA level **(C)**, METTL3, and CCND1 protein level **(D)** were detected after transfected. **(E)** Band density of Western blot was quantified using ImageJ, and protein levels were normalized to β-actin. **(F)** Cellular proliferation was assessed using CCK-8 assays. **(G)** Flow cytometry was used to assess distributions of cells across the cell cycle. **(H)** The qualifications of the cell cycle were presented. *p < 0.05, **p < 0.01, ***p < 0.001.

Recent studies have shown that the m^6^A RNA modification regulated the progress of diverse cancers and thousands of transcripts, including miRNAs (10). The m^6^A methylation level in cervical cancer is significantly higher than the adjacent normal tissues (**Figure 4A**). Three important subunits of the m^6^A methyltransferase complex, methyltransferase-like 3 (METTL3), METTL14, and WT1 associated protein (WTAP), were found different expression in tumor tissues (**Figure 4B**). Among them, METTL3 showed the most different mRNA levels between the normal and cancer tissues (**Figure 4B**). Low miR-193b and high CCND1 were found in low METTL3 patients (**Table 3**). So we performed siRNA-mediated downregulation of METTL3 in Hela and Siha cells. The transfection efficiency was verified by qRT-PCR (**Figure 4C**) and Western blot (**Figures 4D, E**). Increased tumor proliferation (**Figure 4F**) and cell cycle progression (**Figures 4G, H**) occurred as a result of METTL3 inhibition.

**Table 3:** Relation among METTL3, miR-193b, and CCND1 in cervical cancer.

Variable	Cases		High METTL3 expression		Low METTL3 expression		*p* value^a^
	N	(%)	N	(%)	N	(%)	
miR-193b							
High expression	15	36.58	11	26.83	4	9.76	0.017
Low expression	26	63.41	9	21.95	17	41.46	
CCND1							
High expression	21	51.22	7	17.07	14	34.15	0.043
Low expression	20	48.78	13	31.71	7	17.07	

a χ^2^ test.

**Figure 5:**
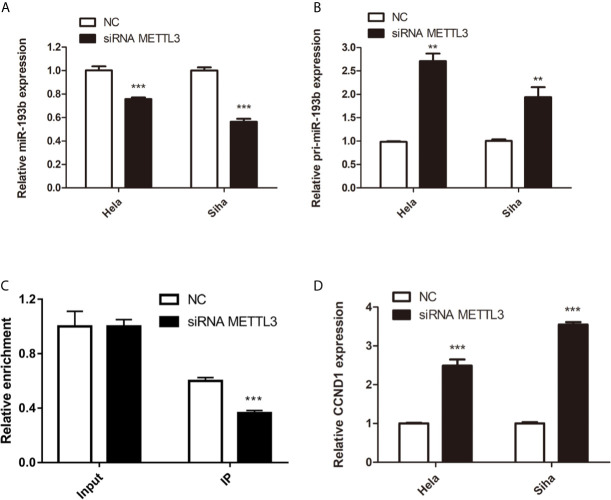
miR-193b is regulated by m^6^A methylation. Hela and Siha cells were transfected with siRNA METTL3 or negative control. Expression levels of miR-193b **(A)**, pri-miR-193b **(B)**, and CCND1 **(D)** were measured by qRT-PCR. **(C)** Immunoprecipitation of m^6^A modified RNA in control or METTL3 downregulated cells followed by qRT-PCR to assess the pri-miR-193b m^6^A modification levels. **p < 0.01, ***p < 0.001.

Silencing METTL3 in cervical cancer cells inhibited miR-193b levels and increased the expression of pri-miR-193b (**Figures 5A, B**), suggesting that METTL3 modulated miR-193b mature process. RIP assays were used to further discover the role of METTL3 in cervical cancer. We found that METTL3 promoted pri-miR-193b m^6^A level (**Figure 5C**). METTL3 siRNA upregulated the expression of CCND1 on both protein (**Figures 4D, E**) and mRNA levels (**Figure 5D**). The results suggest that METTL3 modulates miR-193b mature process by promoted pri-miR-193b m^6^A methylation level.

## Discussion

Tumor biology research has revealed miRNAs to be able to exert potent oncogenic or tumor-suppressing properties (25, 26). This investigation found that downregulated miR-193b was significantly associated with advanced FIGO stage and deep stromal invasion in cervical cancer. As expected, miR-193b served as a tumor suppressor in cervical cancer through induction of cell cycle G1 phase arrest and inhibition of cellular proliferation. miRNAs are predicted to regulate more than 60% of all human mRNAs and are involved in almost all biological processes in mammal systems (27). Functional characterization of miRNA target networks as well as identification of dysregulated miRNA expression has both highlighted the critical role of miRNAs in malignancy (28). Through binding to target sites in mRNA 3’-UTR regions, miRNAs can modulate protein-coding gene expressions (15, 29). Using the miRNA target prediction algorithm, we found CCND1 as one of the miR-193b target genes and verified it in the following experiments.

As a cell cycle regulator, CCND1 is associated with cyclin-dependent kinases 4 or 6 (CDK4/6). The CCND1/CDK4/6 complex phosphorylated the retinoblastoma protein to release the E2F transcription factor that is responsible for triggering G1-S phase cell transition (30, 31). Accumulating evidence demonstrated that cervical cancer specimens harboring raised CCND1 levels were linked to lower overall patient survival (21–24). Interestingly, genetic amplification has been mostly ruled out as a cause of overexpression of CCND1, suggesting the presence of a post-transcriptional process (32–34). Numbers of miRNAs involved in cell cycle control were identified in recent studies. In gastric cancer, miR-218 was noted to inhibit cell cycle progression *via* the CDK6/CyclinD1/E2F1 axis (35). Up-regulation of miR-15a and miR-16-1 induced leukemic cell death by resulting in the downregulation of the target gene CyclinD1 (36). In our study, we indicated that miR-193b not only led to a G1 arrest in Hela and Siha cells through CCND1 targeting but also acted to suppress malignant features of cervical cancer in xenograft models.

The N^6^-position of adenosine methylation is one of the main epigenetic aberrations in the inactivation of tumor suppressors (37). In our study, low miR-193b expression was found in cervical cancer. The miR-193b precursor is located on chromosome 16p13.12, where the loss of heterozygosity (LOH) is rare in cervical cancer (38, 39). Therefore, we hypothesized that the expression of miR-193b was epigenetically silenced by m^6^A. m^6^A mark triggers the formation of miRNAs in a post-transcriptional manner (10). Biogenesis of miRNAs begins when pri-miRNAs are processed by the microprocessor complex, comprising of type III RNase DROSHA and the RNA-binding protein DGCR8 (40, 41). DGCR8 is first required to detect stem junctions in flanking single-stranded RNAs of the pri-miRNA hairpin (42). Subsequently, DROSHA recruiting results in RNA duplex cleaving to yield pre-miRNA (42). During pri-miRNA processing, METTL3, an important component of the m^6^A methylase complex, methylates pri-miRNAs and labels them failure for DGCR8 identification and processing (10). The efficiency of m^6^A mark in regulating pri-miRNA processing has been confirmed in *in vitro* experiments (10). However, relatively little is known about how m^6^A modulates miRNA expression in cervical cancer. As expected, methylation of pri-miR-193b could be detected by using METTL3 siRNA to mediate the loss of miR-193b and the accumulation of pri-miR-193b. Correspondingly, up-regulated CCND1 and suppressed cancer aggressiveness were observed after METTL3 RNAi. The evidence supports the notion that m^6^A modification associated epigenetic silencing of miR-193b occurs during the development and progression of cervical cancer.

In conclusion, our results suggest that cervical cancer may be critically modified by tumor-suppressing miR-193b which is in turn regulated by m^6^A modification. Reintroduction of miR-193b markedly suppressed *in vitro* and *in vivo* tumorigenicity of cervical cancer cells through CCND1 targeting. These findings show that miR-193b may be used as a miRNA-based tumor therapy target in the future.

## Data Availability Statement

The original contributions presented in the study are included in the article/**Supplementary Material**. Further inquiries can be directed to the corresponding authors.

## Ethics Statement

The studies involving human participants were reviewed and approved by the Research Ethics Committee of Sun Yat-Sen Memorial Hospital. The patients/participants provided their written informed consent to participate in this study. The animal study was reviewed and approved by the Animal Research Committee of Sun Yat-Sen University.

## Author Contributions

CH, JL, ZL, and TY designed the experiments. CH and JL finished the first draft of the manuscript. ZL and TY completed the final version. CH focused on the CCK-8 assay, cell cycle analysis, qRT-PCR, Western blot assay, IHC, and Luciferase reporter assay. JL and SL collected tissue samples as well as clinic data, and completed statistical analysis. DW and QX performed most of the animal studies. All authors contributed to the article and approved the submitted version.

## Funding

This work was supported by the National Natural Science Foundation of China (81972433), and the Natural Science Foundation of Guangdong Province (Doctoral Research Start-up Fund Program) (2016A030310178).

## Conflict of Interest

The authors declare that the research was conducted in the absence of any commercial or financial relationships that could be construed as a potential conflict of interest.
