# Supplementary material for: N6-Methyladenosine Associated Silencing of miR-193b Promotes Cervical Cancer Aggressiveness by Targeting CCND1
Source: Front Oncol. 2021 Jun 10;11:666597. doi: 10.3389/fonc.2021.666597 (PMC8222573; doi:10.3389/fonc.2021.666597)
Supplement: Supplementary file 1 [file DataSheet_1.doc]

Fig.S1. The transfection efficiency in Hela and Siha cells. The transfection efficiency of miRNA (A) or anti-miRNA (B) was verified by qRT-PCR. *** p< 0.001.
