# Supplementary material for: N6-Methyladenosine Associated Silencing of miR-193b Promotes Cervical Cancer Aggressiveness by Targeting CCND1
Source: Front Oncol. 2021 Jun 10;11:666597. doi: 10.3389/fonc.2021.666597 (PMC8222573; doi:10.3389/fonc.2021.666597)
Supplement: Supplementary file 2 [file DataSheet_2.doc]

**Supplementary Materials**

**qRT-PCR primers:**

CCND1-F: CGTGGCCTCTAAGATGAAGG

CCND1-R: CTGGCATTTTGGAGAGGAAG

GAPDH-F: GCACCGTCAAGGCTGAGAAC

GAPDH-R: TGGTGAAGACGCCAGTGGA

miR-193b-F：GCCGCAACTGGCCCTCAAAGT

miR-193b-R：GTGCAGGGTCCGAGGT

U6-F: GCTTCGGCAGCACATATACTAAAAT

U6-R: CGCTTCACGAATTTGCGTGTCAT

**siRNA, miRNA mimic and inhibitor sequences:**

CCND1 siRNA

GGAGAACAAACAGAUCAUCTT

GAUGAUCUGUUUGUUCUCCTC

CCND1 siRNA NC

UUCUCCGAACGUGUCACGUTT

ACGUGACACGUUCGGAGAATT

miR-193b mimic

Sense AACUGGCCCUCAAAGUCCCGCU

Antisense CGGGACUUUGAGGGCCAGUUUU

miR-193b mimic NC

Sense GUACCUGACUAGUCGCAGATT

Antisense UCUGCGACUAGUCAGGUACTT

miR-193b inhibitor

AGCGGGACUUUGAGGGCCAGUU

miR-193b inhibitor NC

CAGUACUUUUGUGUAGUACAA

**Primers for reporter vector construction:**

CCND1-uf: ACGTCTAGAtgacctgtttatgagatgctg

CCND1-ur: GATCATATGgggtccaccatggctaagtga

Mu-ccnd1-uf: GCAGAGGATGTTCATAAgctatggcTGATTTATAAATGCAATC

mu-ccnd1-ur: GATTGCATTTATAAATCAgccatagcTTATGAACATCCTCTGC

miR-193b-F: TAGAAGCTTCAGTAACGATTCCTTCAGTGA

miR-193b-R: TATCTCGAGTTTAGTTTTATGGAAAGTAGC
